# Supplementary material for: Do opinion leaders know more? Knowledge accuracy, self-confidence, and media use in agricultural issues
Source: PLoS One. 2026 Jan 23;21(1):e0341457. doi: 10.1371/journal.pone.0341457 (PMC12829936; doi:10.1371/journal.pone.0341457)
Supplement: S1 Table — (DOCX) [file pone.0341457.s001.docx]

**S1 Table. DKE mean values and curve progressions - Interplay of correct knowledge rate and confidence levels in different sample segments (light grey band denotes 95% bootstrap confidence intervals)**

| SAL1 (Statements: 4, 5, 6)  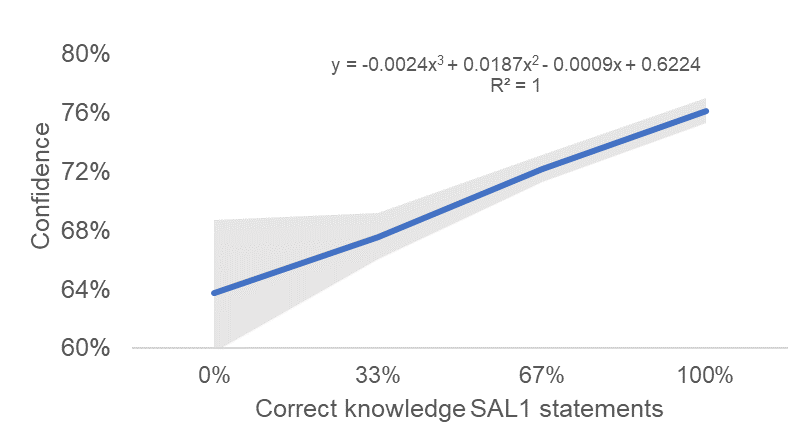 | SAL2 (Statements: 2, 7, 8)  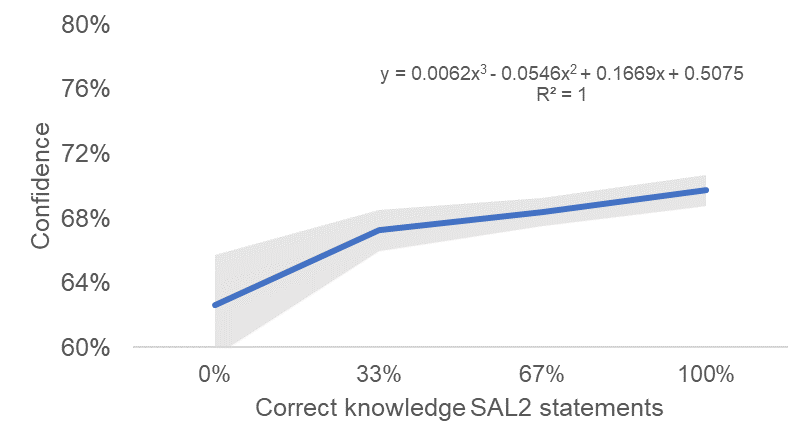 |
| --- | --- |
| SAL3 (Statements: 1, 3, 9)  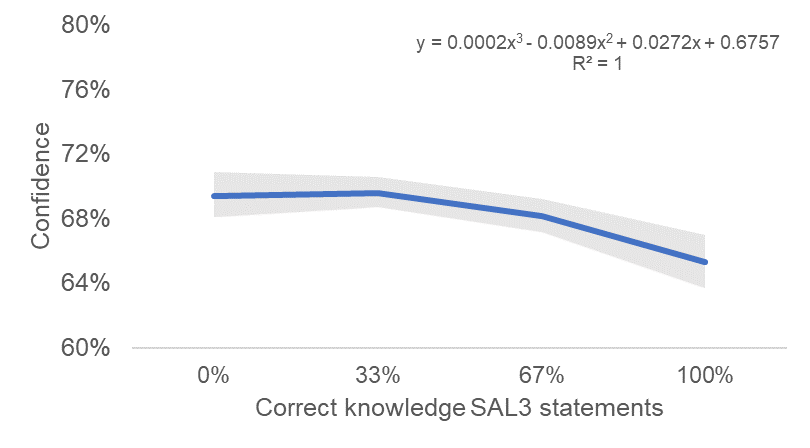 |  |
| False statements (Statements: 1, 3, 6, 7, 9)  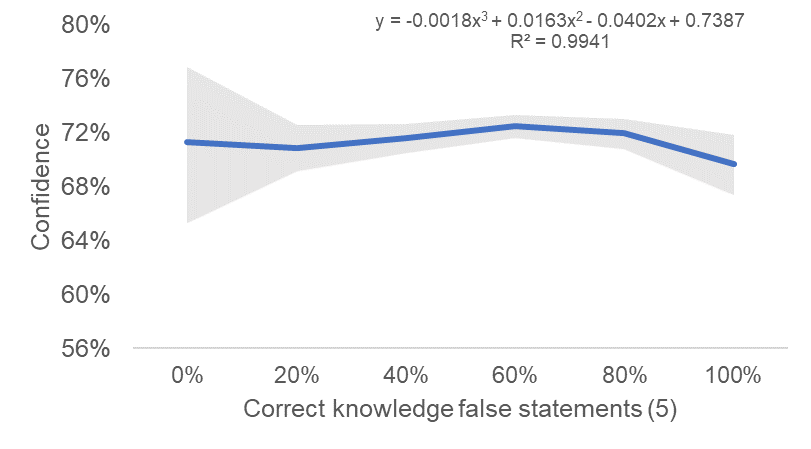 | True statements (Statements: 2, 4, 5, 8)  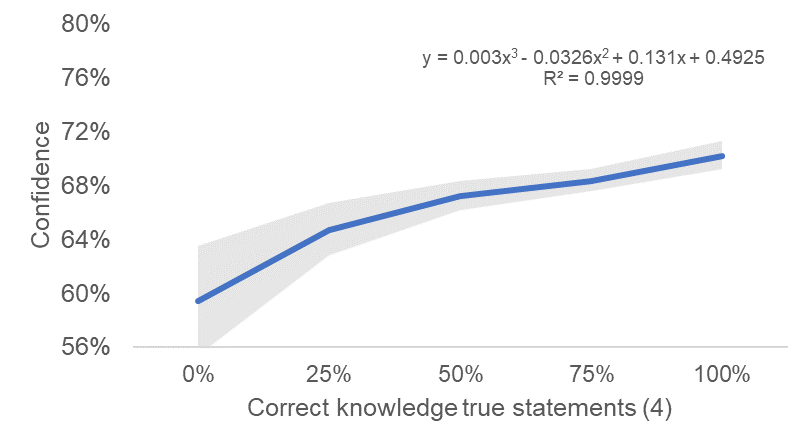 |
| Opinion leader (Q1, n=404)  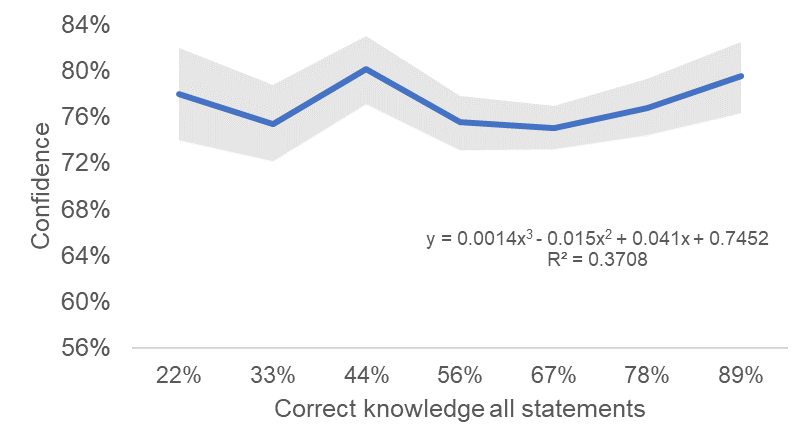 | Strong opinion receiver (Q5, n=404)  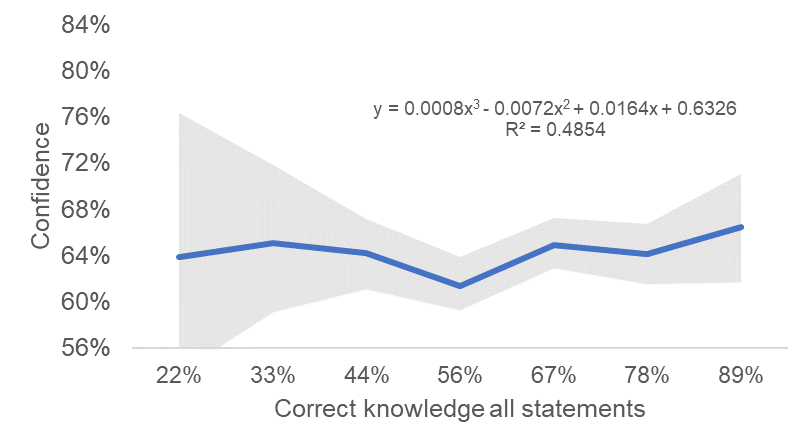 |
