# Supplementary material for: Do opinion leaders know more? Knowledge accuracy, self-confidence, and media use in agricultural issues
Source: PLoS One. 2026 Jan 23;21(1):e0341457. doi: 10.1371/journal.pone.0341457 (PMC12829936; doi:10.1371/journal.pone.0341457)
Supplement: Attachment — Submitted filename: Opinion Leader_Response to Reviewers.docx [file pone.0341457.s004.docx]

We would like to sincerely thank the Editor and the Reviewers for their careful reading of our manuscript and for their constructive and insightful comments. We have carefully considered all suggestions and revised the manuscript accordingly. Below, we provide a detailed, point-by-point response to each comment, including explanations of the changes made and indications of where these revisions can be found in the manuscript.

## Comments of Reviewer #1

***Comment 1.1*** *This research examines the opinion leaders’ characteristics, especially within the agricultural issues, which is truly an important area. However, this paper has critical gaps and severe weaknesses for publication in its current form in the study’s motivation, research question development, literature review, and methodology, especially the conceptualization and operationalization of constructs and the positioning and implication for the study.*

**Response:** We thank the reviewer for this overarching assessment and for highlighting the core areas requiring improvement. We addressed these concerns through a substantial revision of the manuscript. Specifically, we (i) clarified the conceptual positioning of the study and its contribution in the Introduction, (ii) explicitly defined the analytical logic and role of key constructs (knowledge accuracy, self-confidence, media use, and perceived opinion leadership), and (iii) moderated and sharpened the interpretation and implications in the Discussion to align strictly with the empirical scope of the data. These changes are primarily reflected in Section 1 (Introduction, p.8, l.176-186 tracked version), where the research motivation, gap, and objectives are now integrated into a coherent narrative, and in Section 2 (Methods, p. 11, l.251-254) and Section 4 (Conclusions), where construct operationalization and implications are clarified.

***Comment 1.2.*** *First and foremost, the author(s) need to clarify the exact independent variables and dependent variables, and the relationships between them. The title and the abstract are required to be reorganized to describe the IVs, DVs, and their relationship explicitly.*

**Response:** We agree and addressed this comment directly First, in the Introduction, we now explicitly state that perceived opinion leadership is treated as the outcome variable, while knowledge accuracy, self-confidence, media use, and selected sociodemographic characteristics are examined as associated explanatory factors (Introduction, final paragraph before the transition to Methods).Second, the Abstract was revised to clearly describe the relationship between knowledge accuracy, confidence, media use, and perceived opinion leadership, without implying causality. Third, the title was streamlined to reflect the analytical focus on knowledge accuracy and self-confidence in relation to opinion leadership, avoiding ambiguous or causal wording

***Comment 1.3*** *The introduction needs to be rewritten and reorganized. It is unclear what the research gap or research problem is. In particular, social cognitive theory and opinion leadership have been investigated for more than 30 years in many disciplines, including marketing, management, psychology, accounting, finance, and communication. Numerous studies revealed the factors affecting individuals’ attitudinal and behavioral aspects, and the current version of the manuscript does not clearly state the gaps within the literature and how to fill these gaps. What is the ultimate theoretical contribution and the practical implications of this study? I see the great contributions in the findings, but they are not highlighted enough in the manuscript. Why would practitioners, policymakers, and researchers care about your research? The authors should elaborate more on these issues in the Introduction and Discussion sections.*

**Response:** The Introduction was reorganized to foreground the research problem earlier and to clearly specify the gap addressed by this study. Rather than re-testing established theories, the manuscript now emphasizes its contribution in empirically disentangling confidence, knowledge accuracy, and perceived influence within a general-population context for agricultural issues—an area underrepresented in prior opinion-leadership research. Introduction: revised first half (p. 4, l.59-74 – tracked version) and a strengthened articulation of theoretical and applied contributions in the discussion.

***Comment 1.4*** *Theoretical framework. One of my deepest concerns is with the theoretical framework. The author(s) mentioned several research objectives at the end of the introduction part, but why are those objectives important, and how are these objectives associated with the theoretical background? Ultimately, what are the research questions or hypotheses and their directions based on the theory? Social cognitive theory is mainly focused on the macro level discussion and does not provide a specific elaboration of the constructs and variables. The author(s) might want to investigate more modern theories rooted in social cognitive theory such as social identity or identity fusion (Swann, et al., 2014; Swann, Jetten, Gómez, Whitehouse, & Bastian, 2012).*

**Response:** We agree that SCT alone does not fully explain contemporary opinion leadership. The manuscript now explicitly treats SCT and TSFCM as guiding frameworks rather than exhaustive explanatory models and integrates insights from motivated reasoning, identity-related heuristics, and related communication theories to contextualize the findings (see p.7, l.160ff -tracked version) The revised manuscript now includes a more detailed discussion of opinion leadership research, including extensions such as market mavenship, explicitly linking these streams to self-confidence and information sharing behavior (p.6 lines 115-118 -tracked version´& p. 15, lines 343ff). In addition, the role of the Dunning–Kruger Effect is now clearly framed as a confidence-calibration perspective, not as a stand-alone explanatory theory. This integration strengthens the theoretical grounding of the study.

***Comment 1.5:*** *Similarly, a detailed elaboration regarding the opinion leadership literature and DKE should be included in the revised version of the manuscript. Opinion leadership has also been investigated intensively and has several extensions, such as market mavenship (Clark & Goldsmith, 2005; Clark, Goldsmith, & Goldsmith, 2008), which directly examined the self-confidence.*

**Response:** The literature review was expanded to situate opinion leadership more clearly within related constructs such as market mavenship and communicative confidence. The Dunning–Kruger discussion was refined to emphasize confidence miscalibration rather than deficit-based interpretations of cognition. We introduced a paragraph in the introduction king opinion leadership to market mavenship and communicative confidence and refined interpretation of DKE as structured miscalibration in the result & discussion paragraph.

***Comment 1.6:*** *More importantly, please consider how to integrate these two different streams of literature coherently. The current version of the manuscript feels that the arguments are scattered without logical coherence.*

**Response:** We addressed this concern by explicitly integrating the literature streams rather than treating them separately. In the revised framework, opinion leadership research explains who communicates and influences, while confidence calibration (DKE) explains how accurately individuals assess their own knowledge. Social Cognitive Theory provides the overarching structure linking both (Section 1, p. 5–7). This integration resolves the previously scattered argumentation and ensures logical coherence

***Comment 1.7*** *My primary concerns are with the methodology and analysis part. First, the author(s) should state clearly regarding the conceptualization and operationalization of the important constructs. What are the ultimate predictors and dependent variables of the current study? It seems that the author(s) are mainly focusing on the antecedents of the opinion leadership (from table 9), then please add the conceptual and operational definitions of the antecedents and dependent variable using the theoretical background.*

**Response:** We now provide explicit conceptual and operational definitions of all predictors and the dependent variable, including item wording, scales, index construction, and aggregation rules (Section 2.2 and 2.2.1–2.2.2, p. 11–15).This clarification ensures transparency regarding how theoretical constructs are translated into empirical measures.

***Comment 1.8:*** *The rest of the part, except the regression part, seems unnecessary. As the author(s) might know, the correlations did not provide any insights regarding the causal relationship. Normally, from the modeling perspective, correlations among variables, AVE, and VIF should be presented before fitting the regression. Therefore, the author(s) should revise the analysis procedure appropriately following the following process:*

**Response:** We clarify that correlations are used descriptively to establish associations, not causal inference. Regression analysis is presented as the main inferential approach, preceded by diagnostics for multicollinearity and model assumptions (see Methods Section 2.2.4: explicit justification of analytical sequence.)

*Comment 1.9: Again, to what extent are the results found in this research compatible with previous research? I would like to hear the author’s opinion about it in a clearer manner. In terms of practical implications, what is the ultimate recommendation for practitioners? I believe the author(s) can suggest lots of important implications based on the findings, but you need a clear presentation.*

**Response:** We expanded the Discussion to explicitly relate our findings to prior research on opinion leadership, confidence calibration, and media use. In particular, we compare the observed disconnect between knowledge accuracy and perceived influence with established findings from communication and consumer research, and we contextualize the role of overconfidence in light of the Dunning–Kruger literature. Practical implications were refined to avoid overgeneralization and are now explicitly framed as communication- and literacy-oriented rather than prescriptive policy recommendations. (see p.36 l. 825ff; p36, l. 841ff; p.37, l. 850ff, p. 37, l. 857ff-tracked version)

***Comment 1.10****: I wish you the best of luck in pursuing this research and hope my comments are helpful in that regard.*

**Response:** We sincerely thank the reviewer for the constructive feedback and encouragement, which significantly helped to improve the manuscript.

## Reviewer #2

*Comment 2.1 This manuscript addresses an important and timely topic at the intersection of **communication, cognitive biases, and agriculture**. The agricultural sector is highly exposed to misinformation, and the role of opinion leaders in shaping public perceptions is critical both for the farming community and for society at large. The authors should be commended for attempting to bridge fields that rarely meet in empirical work — agricultural knowledge, opinion leadership, and communication theory. This effort has merit and could spark broader debates in agricultural communication and public understanding of science.*

**Response:** We sincerely thank the reviewer for this positive and encouraging assessment of the manuscript’s relevance and interdisciplinary ambition. We appreciate the recognition of the effort to bridge agricultural knowledge research, opinion leadership, and communication theory. This motivation guided the revision process, and we have further sharpened the manuscript to better highlight this interdisciplinary contribution, particularly in the Introduction and Discussion (pp.2-3 and pp. 22-24).

***Comment 2.2*** *That said, while the study is ambitious in its conceptual framing (Social Cognitive Theory, Dunning–Kruger Effect, and the Two-Step Flow of Communication Model), the empirical execution remains modest. The data are based on self-reported surveys with limited knowledge items, and the operationalization of “opinion leadership” is problematic in ways that weaken the conclusions.*

**Response:** We agree with this assessment and have revised the manuscript to ensure that the scope of empirical claims matches the data. We now consistently refer to “perceived opinion leadership” rather than actual influence, and we explicitly discuss the limitations of self-reported leadership measures (Section 2.2.2; page 15, l. 339ff -tracked version). In particular, we clarified that the study captures confidence calibration rather than actual information dissemination or behavioral influence.

***Comment 2.3:*** *In its current form, the manuscript reads as a solid empirical exercise, but one that overstates its implications and does not yet deliver the strong evidence that its theoretical framing promises. I would encourage the authors to simplify their presentation, report their results more rigorously, and be more cautious in drawing societal and policy conclusions*

**Response:** We revised the Discussion and Conclusions to explicitly avoid causal or policy-prescriptive claims. Societal relevance is now framed in terms of communication risks and literacy challenges rather than direct policy effects (see p. 37, l. 855ff; p. 38, l. 896ff; tracked version).

***Comment 2.4****: “The short title is clearer and more effective than the long version.”*

**Response:** We agree and retained the concise framing while streamlining the long title to avoid redundancy

***Comment 2.5:*** *The abstract is descriptive and clear but lacks quantitative information. Including numbers such measures of over- and underconfidence, average correct response rate of 64%, and proportion of overconfident respondents would substantiate the claims*

**Response:** We revised the complete abstract to include key quantitative indicators, such as average knowledge accuracy and the prevalence of overconfidence (Abstract, p. 1–2 tracked version).

***Comment 2.6:*** *The abstract does not sufficiently explain why the findings matter for society. Overconfidence among opinion leaders could translate into misinformation risks and resistance to sustainable agricultural policies, but this connection is only implied, not spelled out.*

**Response:** We agree and strengthened the abstract by explicitly linking overconfidence among opinion leaders to potential misinformation risks and challenges for evidence-based agricultural communication. Now, we explicitly link overconfidence among opinion leaders to misinformation risks and resistance to evidence-based communication in the abstract. (latter part of abstract).

***Comment 2.7:*** *The conclusion of the abstract is weak, falling back on the generic “further research is needed.” A stronger closing would highlight what is at stake if overconfidence among opinion leaders is left unaddressed.*

**Response:** We replaced the generic closing with a more concrete statement emphasizing the risks of unaddressed overconfidence and the relevance for communication strategies. (last sentence of the abstract).

***Comment 2.8:*** *The introduction is educational and well written, but excessively long on theory. Extended explanations of SCT, DKE, and TSFCM are useful for students, but journal readers may find this overwhelming.*

**Response:** We streamlined the Introduction by reducing explanatory passages and integrating theory more directly with the research problem and objectives (pp. 2–5, comparison with original version shows reduction)- Nevertheless, the requests of the other two reviewers to take certain theories and concepts into account were also addressed.

***Comment 2.9:*** *Objectives are only listed at the very end, which dilutes the focus. They should be stated earlier to guide the reader.*

**Response:** We integrated the study objectives into the argumentative flow of the Introduction rather than only listing them (p.8, l. 176ff)

***Comment 2.10:*** *The scope of analysis is modest (agricultural knowledge in Germany), yet the paper mobilizes references from health, climate, nutrition, and food literacy. This gives breadth, but also raises expectations that the empirical part cannot match.*

**Response:** We narrowed the framing language and explicitly contextualized references from adjacent domains as illustrative rather than directly tested (see p.5 l.108ff and section 3.1.3 -tracked version)

***Comment 2.11:*** *Opinion leadership is measured entirely by self-report (adapted Childers scale). This is a serious limitation: people may overestimate their influence, confusing talkativeness or ego with actual impact. Without external validation (peer nominations, network analysis, behavioral measures), the OLI remains a proxy for perceived leadership rather than true influence.*

**Response:** The construct is now consistently labeled “perceived opinion leadership”. We explicitly acknowledge that the Opinion Leadership Index captures perceived rather than objectively validated influence and discuss this as a core limitation. (e.g., p.15, l. 344ff tracked version)

***Comment 2.12:*** *Knowledge measurement is based on only nine true/false statements. While carefully selected, this is a thin operationalization of agricultural knowledge and may not capture complexity.*

**Response:** We acknowledge this limitation explicitly and justify the approach as a trade-off between survey feasibility and measurement breadth (see p. 12, 277ff tracked version)

***Comment 2.13:*** *Reporting issue: the factor analysis reports p = 0.000 (line 268). This is incorrect; p-values are never zero. The proper reporting is p < 0.*

**Response:** Corrected.

***Comment 2.14:*** *The design does not allow testing whether opinion leaders actually spread knowledge or misinformation, nor whether their peers follow them. This gap is critical, given the theoretical framing.*

**Response:** We explicitly acknowledge this limitation and clarify that the study does not measure the actual information disseminated by opinion leaders nor audience reactions or behavioral follow-up. Instead, the analysis focuses on perceived opinion leadership, confidence calibration, and media engagement. This limitation and its implications for interpreting societal impact are discussed in the Discussion section.

***Comment 2.15:*** *The knowledge results are interesting: myths such as “a farmer feeds 1000 people” were among the least correctly answered. This is a clear opportunity for impactful media communication, yet the manuscript does not highlight the public or media resonance such findings could have.*

**Response:** We strengthened the interpretation of low-accuracy items as potential targets for public communication and media literacy efforts (see p. 26, l.597ff & p.38, l. 888ff – tracked version)

***Comment 2.16:*** *Self-confidence results confirm a large share of overconfidence, especially with false statements. However, only a tiny fraction of respondents were well calibrated. This is important, but could be presented more succinctly.*

**Response:** We streamlined the presentation by reducing repetition and emphasizing the key pattern of miscalibration (see paragraph p. 21, l.477ff -tracked version).

***Comment 2.17:*** *Table 9 (predictors of opinion leadership) is one of the strongest parts of the paper. It shows that confidence and media use are much stronger predictors of opinion leadership than actual agricultural knowledge. This is a key finding that deserves more emphasis.*

**Response:** We explicitly highlight Table 9 as a central empirical contribution and emphasize the dominance of confidence and media use over knowledge accuracy (Section 3.3.3 – opening paragraph and revisions in the conclusion section)

***Comment 2.18:*** *Overall, the results are presented clearly, but the connection back to theory (SCT, TSFCM, DKE) is more asserted than demonstrated.*

**Response:** We addressed this by explicitly linking major result back to the relevant theoretical perspective (SCT, TSFCM, DKE) in the Discussion, clarifying what each framework helps explain—and what it does not (p. 36, l. 825ff; tracked version).

***Comment 2.19:*** *The discussion reiterates that opinion leaders are more confident, but not more knowledgeable. This is important, but not new in the broader communication literature.*

**Response:** We explicitly acknowledge that the pattern is not entirely novel but argue that its replication in the agricultural domain and general population context constitutes the contribution. (p. 37, l. 854ff))

***Comment 2.20:*** *The authors extrapolate to policy and education, but the evidence presented is insufficient to justify such broad claims. Without data on what leaders actually communicate or how audiences react, it is risky to infer societal impacts.*

**Response:** We agree and substantially moderated policy and education claims. Implications are now framed as tentative and conditional, aligned with the data’s limits (see Section 4)

***Comment 2.21:*** *The conclusion ends with “more studies are needed,” which weakens the paper’s contribution. A stronger finish would stress the danger of overconfident leaders disseminating misinformation, and the need for targeted literacy efforts.*

**Response:** We revised the conclusion to emphasize the risks of overconfident opinion leaders and the need for targeted communication strategies.

## Reviewer #3

***Comment 3.1:*** *The submission titled “Do opinion leaders know more? Knowledge and self-confidence of opinion leaders and the general population on agricultural issues” is methodologically sound and valuable. I recommend its publication with minor adjustments.*

**Response:** We thank the reviewer for this positive and encouraging assessment. The suggested minor adjustments were carefully implemented and helped to further strengthen the theoretical framing, methodological transparency, and clarity of interpretation.

***Comment 3.2:*** *In the introduction, I suggest modifying the assertion that we are in a time “rife with misinformation” (page 3, line 42). There was a time when we believed the sun revolved around the earth and that diseases were caused by variations in bodily fluids. While it is true that there is always room for further progress, it is important to acknowledge that we have collectively improved our knowledge.*

**Response:** We agree and revised the wording to avoid a historically absolute or normative framing. The text now acknowledges that misinformation is not a novel phenomenon while emphasizing changes in scale, speed, and visibility of information flows in contemporary media environments. (p.4 , l. 66ff – tracked version).

***Comment 3.3:*** *Additionally, I recommend that the authors consult the literature on “motivated reasoning.” This field posits that cognitive biases may be the default mode of thinking rather than biases in the traditional sense. A purely rational cognition, as advocated by Enlightenment philosophers, may be unattainable.*

**Response:** We integrated motivated reasoning as a complementary explanatory perspective, emphasizing that cognitive biases may reflect default cognitive processes rather than deviations from an ideal rationality (see p. 8, l.176ff – tracked version)

***Comment 3.4****: For instance, models in the social communication field, such as the “Third Person Effect” and the “Spiral of Silence”, demonstrate that social acceptance and belonging to social groups may be more significant in decision-making than being correct or scientifically accurate. These evidence-based models can be integrated with the DKE model to provide a comprehensive understanding of self-perception and group behavioral dynamics.*

**Response:** We agree and incorporated these perspectives conceptually to contextualize why perceived influence and social belonging may outweigh factual correctness in communication processes. These models are introduced as complementary lenses rather than as formally tested frameworks(see p. 5, l.108ff – tracked version)

***Comment 3.5:*** *Metanalyses on the Third Person Effect can be consulted for an overview of the topic, not only to strengthen the rationale and introduction but also to further the discussion of the results.*

**Response:** We addressed this suggestion by referencing the Third Person Effect more explicitly in the Discussion to strengthen the interpretation of confidence and perceived influence, while keeping the empirical focus of the study unchanged (see also comment 3.4).

***Comment 3.6****: Figure 1 is not included in the original DKE paper, so it should be contextualized within the broader discussion of the DKE post-publication of the original paper that describes the effect. This is important because it can be perceived as potentially offensive to some due to the Mount Stupid nomenclature. Providing context may help align it with the paper’s broader argument that “understanding-the-DKE-for-improving-knowledge-accuracy” interventions are necessary.*

**Response:** We addressed this comment by clarifying the heuristic and post-publication nature of Figure 1. A contextualizing sentence has been added to the figure caption (page 4, l.77-80 – tracked version), explicitly noting that the illustration is not part of the original Dunning and Kruger (1999) publication and is used solely as a conceptual aid.

***Comment 3.7****: For a reader or researcher from the social communication field, the TSFCM may appear outdated in light of the most current discussions regarding motivated reasoning phenomena. While I do not recommend the authors remove the TSFCM as a theoretical framework, as it contributes to the arguments supporting the methodological choices, I suggest they rephrase it in the rationale to allow for additional evidence-based models for interpreting their measurements. This is because the TSFCM alone cannot seem to explain their results, even when the DKE is added. While it has been documented that individuals with less knowledge overestimate their own knowledge (DKE), the reasons behind our trust in opinion leaders remain unclear, as we do not actively measure their knowledge accuracy in our daily lives. This raises the question of why non opinion leaders overestimate the unskilled. We may rely on heuristics and non-verbal cues to decide whether to agree or disagree with certain individuals (motivated reasoning). Since the TSFCM implies that information nodes possess accurate information (not knowledge), it is somewhat ill-suited to explain the paper’s results alone.*

**Response:** We revised the manuscript to explicitly acknowledge the limitations of TSFCM in explaining complex, polarized media environments. TSFCM is now framed as a foundational heuristic that motivates the focus on opinion leaders, while recognizing that it does not capture competing information nodes or polarization dynamics (see p. 8, l178ff – tracked version).

***Comment 3.8****: Furthermore, addressing DKE as a bias implies the feasibility of an ideal cognition, purely rational, which may not be the case. Heuristics and non-verbal cues can mediate (increase or hinder) social cooperation. DKE may serve as a default feature in cognition, particularly in the context of motivated reasoning, where excessive learning at an accelerated pace could potentially harm ingroup belonging and hinder social cooperation. According to the Third Person Effect we also overestimate agreement with ingroup peers when people may not agree on the topic at hand at all.*

**Response:** We agree and revised the discussion to avoid framing DKE as a simple deviation from rationality. Instead, confidence miscalibration is discussed as a structural feature of human cognition, particularly relevant in socially embedded communication contexts (see p. 36 , l 827ff – tracked version).

***Comment 3.9:*** *Regarding the objectives, I propose reordering them. The primary objective seems to be to “Understand the relationship between knowledge, confidence, and opinion leadership to identify indications for developing communication strategies that enhance public understanding and behavior on agricultural issues” The subsequent three objectives are specific objectives within the main general one, necessitating their placement afterward.*

**Response:** We reordered and integrated the study objectives into a single coherent paragraph, beginning with the overarching objective followed by the specific sub-objectives. (revision of the opbjective paragraph; p. 8, l.178ff – tracked version) ).

***Comment 3.10:*** *Regarding the methods, given that a consumer panel was assessed, it is crucial to address selection bias as a potential study limitation. Respondents in the consumer panel were not only more inclined to participate in surveys but may have also been primed to survey methods and study designs prior to participating in the reported study. While this does not invalidate the paper’s results (methodological choices were highly innovative and appropriate), it is a consideration that future readers of the final published research report should take into account.*

**Response:** We explicitly acknowledge potential selection bias associated with consumer panel participation and discuss its implications for interpretation and generalizability (see revision of section 2.1)

***Comment 3.11:*** *All validation indicators mentioned on page 7, line 153, should be thoroughly described in the supplementary material or in the methods section. Assessing internal validity is also part of the methods and should be adequately described for reproducibility purposes.*

**Response:** We clarified the validation procedures used and explicitly state their role in ensuring internal consistency and plausibility of responses (p.10, l.218ff – tracked version)

***Comment 3.12:*** *Table 1 should be placed in the Results section, not in the Methods.*

**Response:** We respectfully retain Table 1 in the Methods section. The table serves to describe the sampling procedure and to contextualize the study sample relative to the general German population, which is an essential component of the study design. The stratification variables presented in Table 1 (e.g., age, gender, residential area size) are introduced here because they inform both representativeness and subsequent analytical decisions. These characteristics are later incorporated as explanatory variables in the analyses rather than being treated as outcome-related results. Placing the table in the Methods section therefore aligns with its function as a description of the sampling framework rather than a substantive empirical finding. This placement is consistent with common reporting standards in survey-based research, where sample composition is reported as part of the methodological context.

***Comment 3.13:*** *Age was associated with not participating in opinion leadership, and the sample is older than the average population of Germany. People under 30 and the wealthy are underrepresented, and this should be discussed in light of the findings in the discussion section. If this deviation is relevant to the findings, if not, and to what extent.*

**Response:** We acknowledge this point and agree that age-related differences in opinion leadership are an important consideration. The slight overrepresentation of older respondents and the underrepresentation of younger and higher-income groups are inherent to the sampling structure of large-scale online panels and are already reflected in the descriptive statistics. Age is explicitly included as an explanatory variable in the regression analysis, allowing its influence on opinion leadership to be statistically accounted for. Given that the main conclusions of the study are robust across age groups and that age does not alter the central pattern of confidence-driven opinion leadership, we did not further expand the discussion on this aspect to avoid overstating subgroup-specific effect.

***Comment 3.14****: In page 9, line 185, it is important to explicitly outline the approach in the text as well as referencing it (ref 39) for transparency and reproducibility. If any deviation from the approach was carried out, they must also be described along with the rationale for such a deviation from the reference. If no deviation took place, that should also be explicitly stated*

**Response:** We appreciate this clarification request. The analytical approach applied in this study follows the procedure described in ny Thaller and Brudermann without deviation. All steps, including the construction of the knowledge items, the confidence scale, and the calculation of calibration measures, were implemented as specified in the referenced approach. As no methodological adaptations or deviations were introduced, we did not further elaborate on this aspect in the manuscript in order to avoid redundancy

***Comment 3.15:*** *Also in page 9, how many experts were outsourced, and based on what criteria were they recruited? Who was responsible for the curation and wording of the final set of questions, and were they also experts on agricultural topics? As the DKE seems to be sensitive to question framing, questions should be relevant and addressed in the methods section.*

**Response:** We clarified the expert involvement, selection criteria, and responsibility for final wording (see page 12, l. 267ff – tracked version)

***Comment 3.16:*** *In page 11, I agree with the usage of the equation (reference 45), but it is important for the authors to describe what the equation was originally used for and to what each notation originally corresponded with so that the reader may understand the thought experiment rationale of redeployment of the equation for the study’s purpose.*

**Response:** We added a brief explanation of the original purpose of the equation and clarified its adapted use in the present study. (p. 15, l.326ff- tracked version 1).

***Comment 3.17:*** *In page 12, line 248, how was the internal validity of the scale tested in the context of the present study? This should be detailed in the methods section.*

**Response:** We added a brief explanation of the original purpose of the equation and clarified its adapted use in the present study (p. 16, l.356-358; tracked version)

***Comment 3.18:*** *On page 13, line 274, it is stated that “Several multivariate methods were used to analyze the interactions.” However, have all of these choices been described in the methods section? Please address those gaps*

**Response:** We ensured that all multivariate analytical steps referenced in the Results are explicitly described in the Methods section.

***Comment 3.19:*** *On page 19, lines 413 and 414, these results can be thoroughly explained by the Third Person Effect.*

**Response:** We agree that the observed self–other assessment pattern aligns well with the Third Person Effect. To make this theoretical linkage explicit, we added a brief reference in the discussion of opinion leaders’ self-assessment relative to the general population (seep. 33; l. 750ff – tracked version)

***Comment 3.20:*** *Figure 4 should deploy fixed proportions for the horizontal axis, specifically 20, 30, 40, and 50, for the purpose of scale interpretation. The vertical scale may maintain intervals of 4 points as they are consistent.*

**Response:** We agree and revised Figure 4 to use fixed horizontal axis proportions (20, 30, 40, 50) while maintaining consistent vertical scaling

***Comment 3.21:*** *On page 22, line 485 is missing the term “internet sources.” Line 487 must have the aggregate proportion corrected from 40% to 61% (for newspapers) as the brackets described are both “rarely” and “never.” On the same page, in line 491, it is best to use “around” instead of “over” for accuracy. That is, where it reads “while over half never consult them,” it should read instead “while around half never consult them.”*

**Response:** corrected

***Comment 3.22:*** *On page 25, line 556, instead of “-0,096,” it should read “-0,092” according to the results displayed in table*

**Response:** corrected

Comment 3.23: *Although the supporting data has been made available, a supplementary file explaining the column labels is needed.*

**Response:** We agree and have addressed this point by adding a dedicated tab for variable and column labeling in Supplementary Material S2 (Tab ‘Variable_Label’). This tab provides a structured labellng of all variables used in the supporting dataset, including their meaning, scale, and coding, thereby improving transparency and reproducibility
